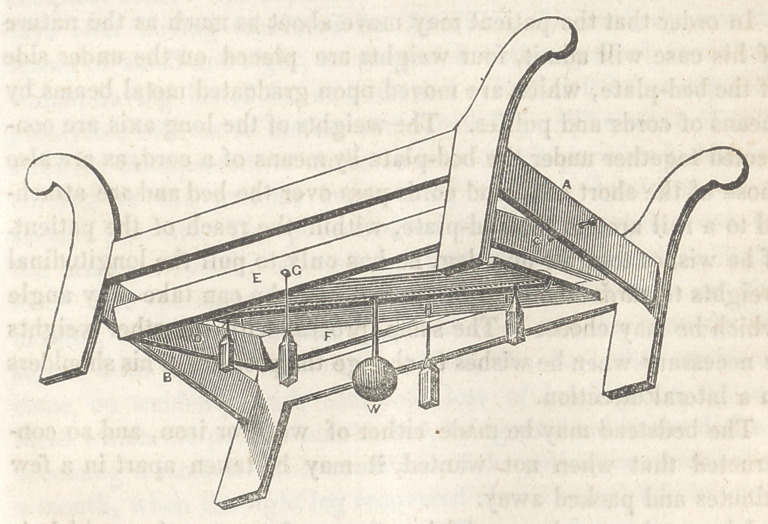# Description of a Bedstead for the Treatment of Fractures of the Lower Extremities on Board Ship

**Published:** 1849-06

**Authors:** W. F. Jackson

**Affiliations:** Brunswick, Maine


### MEDICAL EXAMINER,

AND

### RECORD OF MEDICAL SCIENCE.

NEW SERIES.—NO. LIV.—JUNE, 1849.

### ORIGINAL COMMUNICATIONS.

*Description of a Bedstead for the Treatment of Fractures of the Lower Extremities on board ship.* By W. F. Jackson, M. D., Brunswick, Maine. (Communicated in a letter to Professor Mütter.)

*Dear Sir,*—In accordance with your request, I send you a description of the "Fracture Bedstead," the model of which I recently exhibited to you; and the same excuse which I then offered for presenting so rough a model, must answer for the no less rough description. I need say nothing of the design of the bedstead, nor of its applicability to its object, for you are already acquainted with the former, and are, of course, a far better judge of the latter than myself.

This apparatus consists of a common bedstead, or one modified to suit the taste of the individual. Through the centre of the cross rails A B, pass two centres which are screwed into the rails, and which support the frame C, D, E, F, at the joints C, D. By this means, the frame is allowed to play freely upon its longitudinal axis, and thereby avoid the *rolling* motion of the ship, provided that the bedstead is placed " fore and aft." Within this frame is placed the bed-plate, which is supported by means of centres at the points G, H. The bed-plate will therefore counteract the *pitching* of the ship. If the bedstead be placed "athwart ship," the actions of these planes will of course be reversed.

In order to make the bed-plate maintain its horizontal position, a heavy weight W. is attached firmly to its centre, on the under side, by an iron rod, and this rod must be as long as the height of the bed-plate will allow, so as to get as great a leverage as possible.

In order that the patient may move about as much as the nature of his case will admit, four weights are placed on the under side of the bed-plate, which are moved upon graduated metal beams by means of cords and pullies. The weights of the long axis are connected together under the bed-plate by means of a cord, as are also those of the short axis, and cords pass over the bed and are attached to a rail around the bed-plate, within the reach of the patient. If he wishes to draw up a leg, he has only to pull the longitudinal weights towards the foot of the bed, and he can take any angle which he may choose. The same process with the other weights is necessary when he wishes to change the position of his shoulders in a lateral direction.

The bedstead may be made either of wood or iron, and so constructed that when not wanted, it may be taken apart in a few minutes and packed away.

I have also another model in process of construction, which is somewhat simpler than the one which I exhibited to you, but it requires a little more room. In this case, the patient himself becomes the centre weight, by having the centres of the frame C, D, E, F, placed above him. I think that I might make an improvement in the bedstead, but you are well aware that a student finds but little time for matters not directly connected with his studies, and I must defer it for the present.

Yours respectfully,

W. F. JACKSON.

## Figures and Tables

**Figure f1:**